# Transnational migration, changing care arrangements and left-behind children's responses in South-east Asia

**DOI:** 10.1080/14733285.2015.972653

**Published:** 2014-10-23

**Authors:** Lan Anh Hoang, Theodora Lam, Brenda S.A. Yeoh, Elspeth Graham

**Affiliations:** ^a^ Asia Research Institute, National University of Singapore, Bukit Timah Campus, 469A Tower Block, #10-01, Bukit Timah Road, Singapore258770, Singapore; ^b^ School of Social and Political Sciences, The University of Melbourne, VIC3010, Australia; ^c^ Department of Geography and Sustainable Development, University of St. Andrews, Irvine Building, North Street, St Andrews, KY16 9ALFife, Scotland, UK

**Keywords:** left-behind children, care arrangements, agency, transnational migration, South-east Asia

## Abstract

Recent increases in the volume of labour migration from South-east Asia – and in particular the feminisation of these movements – suggest that millions of children are growing up in transnational families, separated from their migrant parents. Drawing on both quantitative and qualitative data collected in Indonesia, the Philippines, Thailand and Vietnam, the study seeks to elucidate care arrangements for left-behind children and to understand the ways in which children respond to shifts in intimate family relations brought about by (re)configurations of their care. Our findings emphasise that children, through strategies of resistance, resilience and reworking, are conscious social actors and agents of their own development, albeit within constrained situations resulting from their parents’ migration.

## Introduction

With increased mobilities in South-east Asia over the last two decades, new streams of migration have evolved, rapidly transforming the lives of millions of individuals and their families while inextricably linking the fates of states and societies together for better or worse. The transnational family consisting of key members dispersed across international borders is becoming an increasingly prevalent household configuration in South-east Asia, a major exporter of labour migrants (Yeoh, Huang, and Lam 2005). As the search for work takes these migrants – many of whom are parents – away from their homes, millions of children are growing up without their father and/or mother's presence, and entrusted to the care of either left-behind solo parents or relatives in their home countries. Gender-differentiated labour migration in response to changing production and reproduction processes worldwide is hence becoming a significant driver of contemporary social transformation in the family and of caregiving arrangements for children, in sending communities. The migration of either parent, particularly mothers, generates varying degrees of ‘displacement, disruptions and changes in caregiving arrangements' (Scalabrini Migration Center 2004, 61). As individual members rework their roles and responsibilities within the framework of changing family circumstances, children also experience these changes in material, social and emotional terms and respond to the way daily arrangements and relationships of care are reconfigured. Much of the past literature on the transnational family and long-distance parenting has focused on adult actors. In this paper, we engage with the experiences of children ‘left-behind’ and their active involvement in the processes of reconfiguration.

Drawing on findings from a project investigating *c*hild *h*ealth *a*nd *m*igrant *p*arents in *S*outh-*E*ast *A*sia (henceforth referred to by the acronym CHAMPSEA), we examine shifts in care arrangements for left-behind children and the ways in which children respond. We first draw on the findings of large-scale quantitative surveys in Indonesia, the Philippines, Thailand and Vietnam to examine care arrangements in transnational families following the migration of one or both parents, before building on Katz's (2004) work on children's agency in discussing how left-behind children perceive their changing circumstances due to parental migration, and their strategies and responses toward the everyday practices of care.

## (Re)configuring care for left-behind children

Though labour migration is often rationalised as ‘for the family's sake’, the absence of a parent necessarily changes the pattern of everyday life and care for those left behind. Family structures may be altered as tasks are reassigned and existing roles of remaining family members change to fill the gap left by the absent migrant (Gamburd 2000; Hugo 2002; Parreñas 2005a). The type and degree of adjustment required and the capacity for change depend on a variety of factors, including the gender and social class of the migrant and sociocultural norms, as well as other ‘lines of influence' that ‘indicate and highlight what are regarded as the pivotal issues' (Rigg 2007, 175). This section first examines the changing permutations of care arrangements (i.e. mother-carers, father-carers and other carers such as grandparents, aunts and older siblings) within households in Asia^1^ when fathers and/or mothers migrate, and the potential impact on remaining family members. We also seek to locate children's agency in their reactions to and negotiations of changing arrangements for their care.

When fathers migrate, mothers are commonly observed to continue in their socially inscribed role of carers and nurturers of their children while maintaining the existing nuclear family structure.^2^ Though there are seemingly fewer ostensible changes in caring arrangements, taking over the tasks traditionally performed by men adds to women's physical, economic and emotional stress, and may compromise the quality of care left-behind mothers provide. While children appreciate and acknowledge the extension of their migrant fathers’ breadwinning role overseas,^3^ they too may experience negative outcomes not only because of the poorer quality of care they received, but also because in some cases they have to shoulder the burden of assuming extra agricultural/household tasks in their father's absence. Examples of negative outcomes from the CHAMPSEA study include Indonesian and Thai children of migrant fathers being more prone to poor psychological well-being (emotional and conduct disorders, respectively) as compared to children of non-migrant (NM) parents (Graham and Jordan 2011). Elsewhere, Parreñas (2005a, 67) describes Filipino children in her study as suffering an ‘emotional gap’, or some sense of ‘social discomfort and emotional distance' in their relationships with their migrant fathers. Ironically, many of these children – while believing that the relationship can be repaired – actually prefer to spend less time with their fathers on their return, persisting instead in feeling awkward and embarrassed.

Greater changes in living and care arrangements are often observed when mothers migrate. Many left-behind fathers seek the help of extended family members – often female – or even friends and neighbours to undertake caring or nurturing tasks vacated by absent mothers (Gamburd 2000; Parreñas 2005a; SMC 2004). These ‘other mothers’ (Schmalzbauer 2004) absorb some or all of the migrant mother's duties of providing emotional support and physical care to those left behind. Extended households may hence help fill the care deficit created by the migrant's absence (Bruijn et al. 1992) and contribute to the success of migration for the entire family. The existing literature, however, provides a fairly negative view of substitute care arrangements for left-behind children in their mother's absence. Parreñas (2005a, 121) reports that children maintaining ‘biological-based views on mothering' may regard the substitute work of these other relatives as inadequate. Despite the best efforts of extended kin, mothers’ (more so than fathers’) absence may still produce detrimental outcomes for remaining children. Other studies suggest a discouraging scenario especially for left-behind daughters who cope by leaving school earlier and forming their own families through early marriages and unplanned pregnancies in order to fulfil their own lives (Gamburd 2000; Parreñas 2005a). Eldest daughters may assume heavier burdens when their mothers are away as they are called upon to become surrogate parents to their siblings, do housework, make decisions and care for the general well-being of their family (while sons may be saddled with more responsibilities when their fathers leave) (Parreñas 2005a). Some, especially those from poorer families, experience falling grades and a significant decrease in the quality of life after their mothers leave even as they pick up new skills and become more independent. Overall, while some girls resent the heavier burden, others accept it and readjust their daily lives in order to ‘repay’ their migrant mothers.

Evidence from existing studies indicates that left-behind men in countries such as Bangladesh^4^, Indonesia, the Philippines and Sri Lanka do take on *more* caregiving roles when their wives migrate (Asis et al. 2004; Chantavich 2001; Gamburd 2000; Hoang and Yeoh 2012; Hugo 2005). Fathers have also been identified as the main carers by around half of the sample (*n *= 1443 children of migrant and NM parents) of Filipino children of migrant mothers in a 2003 study conducted by the Scalabrini Migration Center in Manila (Asis 2006), and they constitute the majority (over 80%) of main carers in Hugo and Ukwatta's (2010) study (*n *= 400 female migrant households) in Sri Lanka. However, several studies also suggest that such changes are not always sustained or continued after the women return, especially in the Philippines (Afsar 2005; Chantavich 2001; Hugo 2005; Parreñas 2005a). Interestingly, though Sri Lankan men do not openly admit to the assumption of household and child-rearing tasks – work that is seen to threaten their sense of masculinity – Gamburd (2000) observed that there was actually more male participation in housework than reported. These changes in fathering roles may help counteract any negative impact of mothers’ absences on children as most left-behind children view the changes in their father's roles positively. Conversely, children may become confused or resentful of fathers who shun nurturing roles and generally fail to ‘reconstitute fathering in ways that balance and reciprocate the efforts of mothers to perform transnational mothering' (Parreñas 2005a, 140). Children may be upset with fathers who resist carework and gradually become withdrawn due to the lack of emotional support. In addition, left-behind fathers appear to be experiencing greater stress in this reversed situation, with more of them picking up drinking and drug-taking habits as a form of escape (Hewage, Kumara, and Rigg 2011). This may eventually increase risks among children, or have an adverse effect on their behaviour, emotions and performance in school (Gamburd 2005; Hewage et al. 2011; Hewage, Kumara, and Rigg 2011; Hugo and Ukwatta 2010; Senaratna 2012; Ukwatta 2010).

In Asia, many grandparents also act as carers of left-behind children when one or both parents are away. Their presence in the family prior to migration may help ease the reconfiguration of care when a parent, particularly the mother, leaves. They also serve as essential substitute carers when left-behind fathers fail in their caregiving roles (Senaratna 2012). Nevertheless, the limited studies on grandparent-carers in Asia hint at an arrangement that is potentially fraught with tensions and negotiations, sometimes leading to a restructuring or weakening of ties, and estranged relationships between the older generation, their migrant adult children and their left-behind grandchildren (Bruijn et al. 1992; Gamburd 2000; Handapangoda 2012; Hoang, Yeoh, and Wattie 2012; Hugo 2002). As evidenced in Gamburd's (2000) study in Sri Lanka, conflicts between migrants and their own parents or parents-in-law often arise when remittances are disrupted or there are disputes over differences in caring practices between the generations.

Not all the literature points towards negative outcomes, or ‘care deficits’, as a result of parental migration. Zimmerman, Litt, and Bose (2006, 19) remind us that the various support networks mentioned earlier may in time replace ‘a mother's hands on caregiving' and stimulate the development of new care networks and relations. Left-behind children may also grow in their relationship with their surrogate carers and continue functioning well in their daily activities. Indeed, evidence from the CHAMPSEA study suggests that children do adjust to their new circumstances, with those who had spent a larger proportion of their lifetime separated from their migrant mother being more likely to declare themselves ‘happy’ compared to their peers whose mothers had been away for a shorter time (Jordan and Graham 2012). Further, much of the literature on ‘long-distance parenting’ (mainly mothering) shows that caregiving continues even when the parent is physically away. Asis (2002) and Parreñas (2005b), for example, report that the majority of Filipina migrants in their respective studies worked actively at staying connected to their children through phone calls and other means of long-distance communication, succeeding in keeping their families physically and emotionally intact through the period of their absences. Overall, Filipino mothers continue to bear much of the responsibility for childcare and household finances even after leaving the country (Asis 2006; Parreñas 2002, 2005a, 2005b). Children usually appreciate their mother's efforts, and may even feel sorry for them and resolve to behave well and study hard in order to compensate their mothers for their sacrifices. Whatever the responses, the engagement of different family members in practices which keep the transnational family together should be regarded as an expression of active agency within the limits imposed by circumstances.

It is clear from the brief review presented earlier that the views and actions of children left behind as a result of parental migration – despite glimpses from the burgeoning scholarship focused on mobilities within adult lifeworlds – have not taken centre stage in most of the research on the effects of transnational migration on families in South-east Asia. While there is definite interest in increasing our understanding of the well-being of left-behind children in the wake of parental migration, much less has been done in recovering their actions and feelings in response to these significant changes in their everyday lives. This gap in scholarship possibly stems from the assumption that children are often powerless and passive actors in migration as a whole (cf Dobson 2009), having no influence on migration decisions despite frequently being the primary beneficiaries or ‘victims’ of migration. Literature on left-behind children and transnational migration from different parts of the world reports a variety of reactions by children to parents’ absence – they appear passive or indifferent, frustrated or resentful towards the migrant parent (Dreby 2010; Menjivar 2000; Parreñas 2005a).^5^ Most of these studies, nevertheless, focus on adolescents, while the scanty research on preadolescents (Bushin 2009; Orellana et al. 2001) has given attention to children's agency in family migration decision-making and not to the ways they, as immobile members of the family, respond to changes in care arrangements following parents’ migration. Although it is well established that children's ability to exercise agency varies with age, there has been limited scholarly engagement with younger children in migration research.

In our attempt to recover the agency of left-behind children in South-east Asia, we turn to a set of concepts used by Katz's (2004) in her powerful work on growing up ‘global’ in rural Sudan. As Katz (2004) argued, children are not passive recipients of global capitalism but are equally engaged in the commonplace processes of social reproduction. Children's everyday responses may be classified in three ways: resilience, as expressed in the small shifts and turns that allow them to manage and adapt to life's changing circumstances; reworking, in the form of more deliberate, practical actions to alter inequalities and make daily lives more liveable; and resistance, or acts invocating ‘oppositional consciousness … to confront and redress' oppressive and exploitative conditions (Katz 2004, 251). In her work on Howa, Katz (2004, 245) demonstrated the resilience of rural Sudanese in remaining rooted to Howa through the ‘spatial extension of older economic practices', and in escaping proletarianisation caused by outmigration and deskilling due to development projects. To further counter deskilling, rural Sudanese actively ‘rework’ by expanding their schooling in preparation for a changed labour market. They also employ acts of resistance to oppose the production regimes that do not live up to expectations but, instead, threaten their very livelihood. The respondents’ chosen strategy is thus plainly dependent on the various conditions at play. As we show later, the three interrelated concepts of resilience, reworking and resistance offer a useful framework for understanding the feelings and actions of the left-behind children in our study.

## Care arrangements for left-behind children in the CHAMPSEA study

As a context for the following discussion, we draw first on findings from the CHAMPSEA study to provide an overview of the care arrangements for left-behind children in the absence of one or both parents due to transnational labour migration. CHAMPSEA is a cross-sectional mixed-method research programme investigating the impacts of parental migration on children less than 12 years of age left behind in Indonesia, the Philippines, Thailand and Vietnam. The four study countries were carefully chosen to reflect a variety of contexts – transnational labour migration from Thailand and Vietnam is male dominated and is a relatively recent phenomenon (largely since the 1990s), while women account for up to three quarters of transnational labour migrants from Indonesia and the Philippines, both of which have been exporting labour on a large scale over a longer timeframe. Two provinces with high incidences of transnational labour migration in each country – namely, East and West Java in Indonesia; Laguna and Bulacan in the Philippines; Lampang and Udon Thani in Thailand and Thai Binh and Hai Duong in Vietnam – were chosen as study sites. Within each selected province, communities outside the main metropolitan areas and varying in labour-export history (long-standing or recent) and/or in their rural/urban character were chosen.

Primary data were collected in questionnaire surveys conducted in 2008 with around 1000 transnational and NM households^6^ in each study country, as well as through qualitative interviews undertaken in 2009 with around 50 study households in each country. For a household to be eligible for the CHAMPSEA study, it had to include a child in one of two age groups (3, 4 and 5 years or 9, 10 and 11 years).^7^ The sampling strategy was designed to capture the diversity of households distinguished by age and gender of the index child (IC)^8^ and parents’ migration status while maintaining a balance between transnational and NM households. In the surveys, we conducted structured interviews with a responsible adult (RA) in the qualifying household, the carer of the IC (if different from RA)^9^ and the IC themselves, if in the older age group. In the qualitative phase of the study, in-depth interviews were carried out with carers plus a small number of children aged 9–11 years in Indonesia and the Philippines (*n* = 16 in each country). The main aim of the qualitative study was to explore care arrangements for left-behind children, as well as intimate family relations within the (transnational) household. We sought to achieve a balanced sample for qualitative interviews in terms of the migrant's gender, IC's gender and age and carer type in all the countries except for Thailand where only a very small number (*n* = 3) of mother-migrant households were identified in the survey.

Descriptive analyses of the quantitative data showed mixed care arrangements for left-behind children when one or both parents migrated. Grouping all four countries together, the survey data showed that mothers were the main carers of children when both parents lived at home (90.0%) or when fathers were away (93.6%) (Table 1). The care arrangements in father-migrant households displayed in Table 2 also appear rather uniform across all study countries – the mother was the main carer of the IC in most households. This arrangement conforms to the pervasive gender norms not only in South-east Asia but also in most societies that assign caring and nurturing duties to women and breadwinning roles to men (Adams and Coltrane 2005; Taga 2005).

**Table 1.** Care arrangements in all CHAMPSEA's households (*n* = 4073).

Carer type	Migration status (%)			
	Father-migrant	Mother-migrant	Both-migrant	Both-UR
Mother	93.6	0.3		90.0
Father		67.8		7.3
Maternal family^a^	2.4	11.7	52.5	0.9
Paternal family^a^	3.2	16.5	40.3	1.4
Other^b^	0.8	3.7	7.2	0.4

^a^Maternal/paternal family includes grandparents and mother's/father's siblings.

^b^Other carers include distant relatives, IC's siblings and domestic workers.

**Table 2.** Care arrangements in father-migrant households (*n* = 1252).

Carer type	Country (%)			
	Indonesia	The Philippines	Thailand	Vietnam
Mother	96.5	91.5	98.8	83.3
Maternal family^a^	1.7	4.8	1.0	1.9
Paternal family^a^	1.2	1.9	0.2	14.0
Other^b^	0.6	1.9	0.0	0.9

^a^Maternal/paternal family includes grandparents and mother's/father's siblings.

^b^Other carers include distant relatives, IC's siblings and domestic workers.

Given the small sample of mother-migrant households in Thailand (*n* = 3), it is excluded from Table 3 which shows that left-behind fathers were the main carers in the majority of mother-migrant households (59.6–70.8%) – a much higher rate than that found by a study of 1200 mother-migrant households in Sri Lanka where fathers only made up 25.9% of primary carers (Save the Children 2006).^10^ However, as studies in various contexts have shown, fathers often enlist extended family members’ help in childcare when mothers migrate, and different family members may therefore be involved in childcare in different ways and to various extents (Afsar 2005; Gamburd 2000; Parreñas 2005a; SMC 2004; Ukwatta 2010).

**Table 3.** Care arrangements in mother-migrant households (*n* = 677).

Carer type	Country (%)		
	Indonesia	The Philippines	Vietnam
Mother	0.7		
Father	67.5	59.6	70.8
Maternal family^a^	15.3	17.0	6.3
Paternal family^a^	12.5	10.6	22.6
Other^b^	4.1	12.8	0.3

^a^Maternal/paternal family includes grandparents and mother's/father's siblings.

^b^Other carers include distant relatives, IC's siblings and domestic workers.

Table 3 also reveals interesting differences in the composition of the category of non-father-carers in Indonesia, the Philippines and Vietnam for mother-migrant households. The difference between proportions of paternal and maternal family members assuming caring roles is not significant for Indonesia and the Philippines. In contrast, the paternal family is clearly more important than the maternal family in Vietnam (22.6% versus 6.3%, respectively) when the mothers are away, underlining the enduring influence of patrilineality and patrilocality norms in Vietnamese society. Of all the non-parent carers in the Vietnamese transnational households, 77% belonged to the paternal side of the family, compared to 34.3%, 24.8% and 35.3% in Indonesia, the Philippines and Thailand, respectively. The role of the Indonesian and Filipino maternal family in childcare appears more prominent when both parents migrate (63.1% and 61.5%, respectively) (Table 4). Differences between Indonesia and Vietnam in the extent to which paternal or maternal families become involved in childcare are a reflection of different family structures prevailing in two societies and have been examined in depth by Hoang, Yeoh, and Wattie (2012). Interestingly, the ‘other carer' category (i.e. older siblings, distant relatives and domestic workers) is not prominent in Vietnam, while it is relatively more important in Indonesia and the Philippines when both parents are away. These differences and similarities among the study countries with regard to care arrangements for left-behind children raise intriguing questions about what underlies the transnational family's childcare choices.

**Table 4.** Care arrangements in both parents-migrant households (*n* = 181).

Carer type	Country (%)			
	Indonesia	The Philippines	Thailand	Vietnam
Maternal family^a^	63.1	61.5	58.6	27.1
Paternal family^a^	26.2	25.6	37.9	72.9
Other^b^	10.7	12.8	3.4	0.0

^a^Maternal/paternal family includes grandparents and mother's/father's siblings.

^b^Other carers include distant relatives, IC's siblings and domestic workers.

## Left-behind children and care relationships in the transnational family

The influence of societal norms on childcare practices in the four study countries revealed by the survey data can also be expected to colour children's responses to the (re)configuration of their care through processes of social reproduction within and beyond the family. It is, nevertheless, important to recognise that children are not only passive receivers of care but are also capable of exercising their own agency, even if limited by their age and circumstances (Graham et al. 2012). To explore preadolescent children's agency in coming to terms with parental migration and the transfer of care following their parents’ departure, we draw on three illustrative case studies from the CHAMPSEA project. Our discussion utilises the framework proposed by Katz (2004) in her study of young people's agency in the context of development and change. As outlined earlier, this comprises three fluid and overlapping sets of responses: resilience, reworking and resistance. In examining the responses of left-behind children in South-east Asia, we establish that young school-aged children are also not truants from adults’ lifeworlds but social actors keenly involved in these same worlds, and seeking to manoeuvre within the constraints imposed on their lives.

### Dat*^11^* – resistance against adults’ care politics

Ten-year-old Dat, along with his 12-year-old mute and deaf sister, lived with their 78-year-old paternal grandfather – Mr Huyen – and an aunt (their father's unmarried younger sister) in Vietnam at the time of the interview in 2009. His father had been sent to a rehabilitation centre after stealing their neighbours’ assets to feed his heroin addiction. Dat's mother had been working in Taiwan for four years, first on a legal contract and later as an irregular migrant after ‘running away’ to work ‘outside’,^12^ which presumably generated a better income. She had no plans to return home in the near future and neither had she been able to visit home because of her undocumented status. She kept in regular contact with Dat through his aunt's mobile phone. No formal childcare arrangement or financial deal was made with regard to caring for Dat when his mother left. Mr Huyen, who was entirely dependent on his meagre income from farming to sustain livelihood expenses, was not consulted about the migration decision despite the fact that Dat's parents were sharing the house with him. The only form of financial support from Dat's mother was payments for the children's tuition fees collected from their maternal grandparents and New Year presents. However, he had never complained about the children's expenses to her simply because, in his words, ‘they are my grandchildren'. As we have discussed elsewhere (Hoang, Yeoh, and Wattie 2012, 739), grandchildren in the patrilineal Vietnam belong to the paternal family which would automatically have the custody of the grandchildren should their parents no longer be available to care for them (Rydstrom and Drummond 2004). However, for many poor elderly grandparents in the countryside like Mr Huyen who have no recourse to regular formal transfers such as pension and state welfare, custody of grandchildren would be considered a burden rather than a blessing, and it is thus not uncommon for the maternal family to step in as surrogate carers. The popular saying ‘*Cháu bà nội, tội bà ngoại*’ (paternal grandma's grandchildren, maternal grandma's burden) expresses a common belief that even though grandchildren belong to the paternal family by name, it is often the maternal family which shoulders the bulk of care work because they are deemed emotionally closer to them (via the mother–child bond).

Struggling with childcare responsibilities and facing financial hardship, Mr Huyen had repeatedly suggested to his daughter-in-law, both directly and indirectly, that the children should be cared for by their wealthier maternal grandparents, an arrangement with which both Dat's mother and the maternal family agreed. However, Dat's maternal grandfather would only take over the care of the children on the condition that Mr Huyen personally brought them to his house. Mr Huyen refused to do so and wanted the maternal family to come to him and formally request his permission to take the children to their place instead. He explains, ‘How on earth can a paternal grandfather send his own grandchildren to their maternal grandfather's care?'^13^ Apparently both sides of the family in this particular circumstance prefer that the children are placed in the maternal family's care, yet neither of them wants to take the first step in the transfer of care for the fear of social criticism due to the violation of the norms of patrilocality and patrilineality. Unable to reach a consensus over the mode of care transfer, Mr Huyen, Dat's mother and the maternal family handed over responsibility for a ‘solution’ to the children by urging them to move to the maternal grandparents’ house on their own accord, or at least take the first step of regularly having lunch with their maternal grandparents, given that ‘their house was closer to the school'. Dat, however, refused to do so:

I told Dat to go to his maternal grandparents’ place to have lunch after the morning class because his [maternal] grandfather has a motorbike to take him back to school [for the afternoon class]. Otherwise, it would be hard for him to walk back here. He did that for a few days but he has been coming home in the past few days. I said to him, ‘I will not cook for you.' He replied, ‘I will cook by myself if you don't'. (Mr Huyen, grandfather-carer aged 78)

Under similar pressure, Dat's sister moved to her maternal grandparents’ house but, for reasons unknown to Mr Huyen, she returned to him after just a few days. Although the children were entirely left out of the migration decision-making process, they were determined to make their own choices with regard to their living and care arrangements, ignoring repeated requests from both sides of the family to move to the maternal grandparents’ place. Having been brought up all their life in the paternal family's house,^14^ the children had grown emotionally attached to Mr Huyen and preferred living with him in poor conditions over the relatively comfortable life that their maternal family could provide. The way Dat and his sister resolutely resisted the care arrangements dictated to them reminds us of Orellana's et al. (2001, 587) comment that children may be able to indirectly assert their preferences by refusing to engage with the adults’ agendas. While such assertions may not always be articulated in vocal form, and in fact may not be understood as rational within adult ways of framing the world, they represent small but effective steps of active non-compliance and agency. In our study, children's ability to make independent decisions appears to be facilitated by their separation from parents – a fact that has also been observed by Dreby (2007) in transnational Mexican families.

### Wasana – resilience in times of adversity

If resilience is defined as ‘achieving desirable outcomes in spite of significant challenges to adaptation or development' (Masten and Coatsworth 1998, 737), Wasana's story reveals a 12-year-old Thai girl's strong resilience in coping with psychological and material difficulties resulting from her father's overseas migration. Wasana was living with her mother, Mrs Duangchan, and a 9-year-old younger brother at the time of the interview. Her father had been working first in Taiwan and then Israel for the past four years. When possible, Wasana's mother undertook low-waged labour in the village but income from this was irregular. The family relied on Wasana's father's remittances to pay for daily expenses and the debt incurred to fund his migration.

Despite growing up in difficult financial circumstances and in the absence of her father, Wasana was constantly the top student in her class. She was well aware of why her father had to be separated from the family and tried her best at school because, for her, it was the only way she could support him:

She is glad because her father's migration means earning money. Occasionally she complains that she misses him and wants him to come back … She wishes her father could stay with her … She is never absent from school. She goes to school every day, never skipping classes even when she is ill…until the teacher orders her to stop. (Mrs Duangchan, mother carer aged 43)

Not only did Wasana work hard at school, she was also keen on helping her mother with housework. Psychological studies have in fact argued that self-regulatory skills,^15^ as demonstrated by Wasana, are a defining feature of youths’ resilience (Buckner, Mezzacappa, and Beardslee 2003; Karoly 1993; Masten and Coatsworth 1998). The only source of encouragement for her to do well at school came from her migrant father during their phone conversations because her mother had neither much schooling herself nor much interest in Wasana's education. Wasana's father used to help her with her studies but since his departure, she had to rely on herself because her mother could not help. Wasana's father would like her to become a teacher or a doctor in the future, but her mother appeared to have little hope that such a dream would come true because she was uncertain if they could afford to pay for Wasana's higher education. At the time of our visit, Wasana's mother had no idea where she would go for secondary school.

Unlike Dat, who was caught in the care politics between his maternal and paternal families, there was no transfer of care in Wasana's case because she had always been cared for by her mother. However, Wasana's father's migration deprived her of the valuable guidance and support in schooling he used to provide for her, while her mother's time was mostly consumed by work and care for Wasana's younger brother who had poorer health and less satisfactory academic performance and behaviour at school. Besides, Wasana appeared emotionally closer to her father and kept ‘complaining' that she missed him while appearing less interested in spending time with her mother. This sentiment was mutual because according to Wasana's mother, her father cared more about the girl than the boy: ‘He loves the girl … When he phones me, he says my daughter and your son'. Through considerable self-discipline and by drawing on the long-distance encouragement of her physically absent parent, Wasana actively adapted and managed challenging circumstances to hold on to the ambitious plans she has for herself to excel in school. The positive tone in Wasana's story stands in contrast with accounts of relationship fracture and emotional gap between migrants and their children reported by studies in other contexts (Dreby 2007; Parreñas 2005a), although it remains to be seen whether Wasana's close relationship with her migrant father endures during her teenage years.

### Calvin – reworking family bonds and the notion of ‘family’

Calvin from the Philippines was left behind by both parents who migrated to France when he was one and a half years old. Ten-year-old Calvin was living with his aunt (mother's sister) – Ana (whom he called Mommy), Ana's son and his older sister at the time of the fieldwork. Unlike his older sister who was close to their parents, Calvin did not have any significant emotional attachment to them which, according to Ana, was due to the fact that his parents left him when he was just a toddler:

They came back last December … this was their first visit since they left nine years ago … Once, his mother saw that he was going to school and prepared breakfast for him but Calvin did not eat it. He asked ‘Whose food is this?' He kept complaining that this was not the same as the one Mommy prepared … She [Calvin's mother] was hurt, although she didn't verbalise it … He just said Mommy does not cook this way … The boy said he did not want to go out with them even though we told him that he had to. Sometimes, when they went out, I would go with them so that he came also … His father is a bit strict so he does not want him to come home. He says never mind, don't come home anymore. (Ana, aunt carer aged 49)

With the help of his cousin – Ana's son – and funding from his parents, Calvin even ran his own computer shop. He was a bright student and won first prize in a school competition in computer science. His parents were applying for a visa to bring him to France but Calvin was not interested. Ana was close to Calvin and described him as a very sweet and obedient boy. Calvin was very loving and caring towards Ana, while his conversations with his parents tended to focus solely on gadgets and he would lose interest if his parents switched to other topics. He even refused to speak to his parents sometimes.

Calvin's story relates to Parreñas’ (2001, 116) observation that there is ‘a sense of social discomfort and emotional distance' between Filipino migrant fathers and their left-behind children. Dreby (2007) also found that migration, especially that of mothers, took an emotional toll on Mexican children who responded to parent's absence by naming carers as mothers, feigning indifference about parents, disregarding parental authority and appearing reluctant to migrate. However, while Parreñas (2005a) reports feelings of loneliness, vulnerability and insecurity among children separated from their parents, Calvin appeared in his carer's account to be enjoying life with his adopted family and unbothered by his parents’ absence. Calvin's responses should be put in perspective as it has been suggested by other research that the effects of parental migration on children are usually more pronounced in adolescence (cf Artico 2003; Dreby 2007; Smith, Lalonde, and Johnson 2004) and are dependent on the quality of care the child receives from his surrogate carer as well as the age of the child when first separated from his parents (Hoang and Yeoh 2012). Nevertheless, Calvin was not a passive care recipient. Instead, he actively sought to be accepted into his carer's family and to establish an emotional bond with Ana:

Calvin is very caring. The last time I was sick, he went to his room to get dressed but before he left, he came to me and said, ‘Mommy, get well soon'. Later he returned to the room to pick up something else and again he came up to me and said, ‘Mommy, get well soon'. Sometimes, he would just enter the room, bringing me some water, medicine, anything that I need. I am really pleased with this boy … while we are chatting [with Calvin's parents], he would sit on my lap, hugging me and constantly kissing me. His mother would say ‘You are with your Mommy again and you keep kissing her.' Calvin would reply ‘You are just jealous.' He is a sweet boy. (Ana, aunt carer aged 49)

Calvin did not only try to bond with Ana. Ana's son, who was eight years older than Calvin, first became jealous when his mother took Calvin and his sister into his home. Calvin, however, gradually won his cousin's heart and had become close to Ana's son by the time of the interview. He slept with his cousin every night, played computer games with him and, later on, shared responsibilities in running two computer shops. Unlike his older sister who kept looking forward to being reunited with their parents, Calvin considered himself as belonging to his adopted family and refused to migrate to France unless he was accompanied by Ana and her son saying, ‘I will go there only if Mommy and my cousin go there with me'. Through a number of small but deliberate steps – from building strong bonds with his surrogate family to demonstrating entrepreneurial success in setting up his own computer business – Calvin is able to counter adult plans made on his behalf to carve out a pathway for his own future.

## Conclusion: revisiting children's agency in parental migration

In her study of Mexican transnational families, Dreby (2007) argues that children left behind may be both powerful and powerless, whether as intended recipients of the benefits of migration or as independent agents with divergent needs that are intensified by the separation from parents. As ‘powerless’ members of their families, children have little influence over the migration decision, yet through their negative reactions to separation, children are able to shape families’ subsequent migration trajectories. Many Mexican parents migrate to work in the USA, where aspirations for family reunification in the destination country are kept alive even in the face of increasingly tight controls on immigration. The contexts in which South-east Asian parents migrate are typically quite different. Rigid migration regimes (fixed-term contract migration with no possibility of family reunion or acquiring citizenship at destinations) mean that many children face long-term separation from their parents and limited scope for shaping future options. Most migrant parents in our study cannot return home in the middle of their contracts to respond to children's needs as did the Mexican parents in Dreby's (2007) study. Neither can the children put pressure on their migrant parents to allow them to migrate as dependents. Given the prevailing migration regime in Asia, unskilled or low-skilled workers are considered ‘guestworkers’ or transient labourers who have no rights to bring along family members and who are expected to return to their home countries when their labour is no longer needed. Nevertheless, as illustrated by our case studies from Vietnam, Thailand and the Philippines, children actively seek to exercise agency within the constrained situations they face.

Although our research focus and methodology are dissimilar to Katz's (2004), the children's responses to care arrangements during parental migration resonate with her concepts of resistance, resilience and reworking, as illustrated by the stories of Dat, Wasana and Calvin. All the children actively and positively acted to shape the major changes in their lives, accommodating themselves to the disruptions caused by migration. Though still at the age when adult care and protection were required on a daily basis, the children were capable of autonomous action. Children's agency, as argued by Orellana et al. (2001), may take particular twists when their relations with adults are situated in transnational social fields. In all the cases we recounted in the paper, parental migration provided the children with the opportunity to take independent action in their lives, albeit to varying degrees. The absence of Dat's parents, for example, allowed him to resist the transfer of care from his paternal grandfather to the maternal family by ignoring repeated requests from both sides, while Wasana tried to show her gratitude to her migrant father's hardship by taking the initiative in school to perform well despite her mother's lack of interest and the absence of her father's day-to-day monitoring and supervision of her studies. Calvin took advantage of his migrant parents’ yearning for his affection and the desire to make up for the lack of parental care to bargain for their financial support so that he could develop his entrepreneurial interests, while at the same time actively cultivating strong familial bonds with his adopted family.

The three case studies presented in this paper are not intended to be representative of our sample and hence cannot speak for all transnational families involved in the study. In the absence of both parents, Dat and Calvin had bonded positively with their substitute carers. While Wasana's caregiving arrangements had not changed, her father's migration had had more of a negative impact. Other children in the CHAMPSEA study responded differently again. Yet the three cases discussed usefully illustrate that children are conscious social actors and active agents in their own development – a fact that has been increasingly recognised in the literature (Ansell 2009; Boyden 2003). Although the children's responses of resistance, resilience and reworking are examined through the lens of their carers, the exercise of agency revealed accords with another study from the CHAMPSEA project based on interviews with Indonesian and Filipino children themselves (Graham et al. 2012). The cases of the three children, along with data from extensive surveys, raise multiple issues about the Southern end of so-called global care chains^16^ that have largely been overlooked in the literature. Firstly, findings from the CHAMPSEA surveys show that care arrangements in South-east Asian societies are embedded in the long-standing norms relating to family structures and relations that need to be taken into account when researching care regimes in the region. Secondly, as Dat's case shows, transfers of remittances should not be viewed as an invariable outcome of labour migration; where there is a breakdown in communication and/or flow of remittances, left-behind children and their carers may find themselves pushed into precarious and unsustainable livelihood situations. Finally, our study suggests that more research needs to be done to understand the multiple ways in which left-behind children exercise agency to sustain, remake or resist the constrained situations created by parental migration, as well as the long-term effects of these processes on these young lives as they transit to adulthood.

## Notes

1 While acknowledging the problems in ‘utilizing the geopolitical imaginary “Asia”' (Pettman 2003, 172), this paper is unable to dwell further on the debates concerning its usage. ‘Asia’ is mainly used here to demarcate the larger political region in which the field sites of this study are situated. The paper selectively reviews the most relevant literature from the larger region (and not merely from the named study sites), given the uneven interests and coverage in this field.

2 This trend has been reported in many Southeast Asian countries such as the Philippines, while exceptions have been observed in some South Asian countries such as Sri Lanka, as well as in Kerala state in India (Desai and Banerji 2008; Gamburd 2000; Gulati 1993; Jejeebhoy and Sathar 2001).

3 If migration is unavoidable, children would often prefer fathers over mothers to migrate (Asis 2006).

4 Bangladeshi husbands purportedly often take on household chores in the absence of their migrant wives with the help of older children (Afsar 2005).

5 We needed to expand our scope here as relevant literature on left-behind children in Southeast Asia remains rather limited as compared to that in other parts of the world.

6 To qualify as a transnational household, parent(s) must have been absent working abroad for a continuous period of at least the past six months. Similarly, a NM household was taken as one in which both parents had been living at the same address as the qualifying child on most nights over the past six months.

7 These two age groups of preschool children and children in middle childhood were chosen to focus available resources and ensure adequate observations in each group to support a range of analyses including comparisons of health outcomes based on biometric measurements.

8 In each household, we focused on one child – referred to as IC – aged either between 3 and 5 years or between 9 and 11 years.

9 As the carer of the IC might neither live in the same household with him/her (e.g. a grandmother living in a different household but providing care for the child during the day) nor have the best knowledge of aspects of the transnational family's life other than childcare, we included the responsible adult (e.g. a resident parent who was not the main carer of the child) in the surveys to obtain information on matters beyond childcare such as remittances and communication.

10 This is lower than in Hugo and Ukwatta's (2010) later study conducted in 2008 where over 80% of Sri Lankan fathers were identified as the main carer in mother-migrant households.

11 All respondents’ names have been changed to protect their anonymity.

12 Our Vietnamese respondents’ term for the unregulated labour market.

13 See Hoang, Yeoh, and Wattie (2012) for a discussion on the complexity of family care politics in Vietnam.

14 The Confucian norm of patrilocality – the co-residence of married couples with the husband's parents – has an enduring influence in the rural North of Vietnam. See Hoang (2011) and Hirschman and Nguyen (2002) for further discussion.

15 Self-regulation refers to those processes, internal and/or transactional, that enable an individual to guide his/her goal-directed activities over time and across changing circumstances (contexts). Regulation implies modulation of thought, affect, behaviour or attention via deliberate or automated use of specific mechanisms and supportive metaskills (Karoly 1993, 25).

16 The concept of ‘global care chains' developed by Hochschild (2000, 131) refers to ‘a series of personal links between people across the globe based on the paid or unpaid work of caring’. Unequal power relations enable countries further up the chain to continue to depend on a pool of cheap (undervalorised) female labour for carework and domestic work.
